# Molecular Mechanisms of Ischemia/Reperfusion Injury and Graft Dysfunction in Liver Transplantation: Insights from Multi-Omics Studies in Rodent Animal Models

**DOI:** 10.7150/ijbs.109449

**Published:** 2025-02-24

**Authors:** Zhengtao Liu, Jun Xu, Ting Que, Shuping Que, Luca Valenti, Shusen Zheng

**Affiliations:** 1 Key Laboratory of Artificial Organs and Computational Medicine in Zhejiang Province, Shulan International Medical College, Zhejiang Shuren University, Hangzhou, China.; 2 Shulan Hospital (Hangzhou), Hangzhou, China.; 3 NHC Key Laboratory of Combined Multi-Organ Transplantation, Key Laboratory of the Diagnosis and Treatment of Organ Transplantation, First Affiliated Hospital, School of Medicine, Zhejiang University, Hangzhou, China.; 4 Key Laboratory of Organ Transplantation, First Affiliated Hospital, School of Medicine, Zhejiang University, Hangzhou, China.; 5 Division of Hepatobiliary and Pancreatic Surgery, Department of Surgery, First Affiliated Hospital, School of Medicine, Zhejiang University, Hangzhou, China.; 6 Birth Defects Prevention and Control Institute, Maternal and Child Health Hospital of Guangxi Zhuang Autonomous Region, Nanning, China.; 7 DingXiang Clinics, Hangzhou, China.; 8 Department of Pathophysiology and Transplantation, University of Milan, Milan, Italy.; 9 Precision Medicine, Biological Resource Center Unit, Fondazione IRCCS Ca' Granda Ospedale Maggiore Policlinico, Milan, Italy.

## Abstract

Rodent ischemia-reperfusion injury (IRI) and liver transplantation (LT) models play crucial roles in mimicking graft injury and immune rejection, developing therapeutic approaches, and evaluating the efficacy of treatments. The application of integrated multi-omics data and advanced omics techniques like single-cell RNA sequencing in rodent models has expanded researchers' perspectives on pathophysiological processes in LT settings. This review summarizes key molecules and pathways associated with reperfusion injury and prognosis in LT models, highlighting the potential of omics data in understanding and improving transplant outcomes. In addition, we highlight the current challenges and future approaches for the application of omics data in rodent LT models. Cross-species validation with human data will improve therapeutic potential. Finally, further applications combining advanced single-cell, spatial omics technologies and machine learning algorithms will help to identify the key regulatory networks in specific cell populations underlying poor outcomes after LT.

## Introduction

Liver transplantation (LT) is considered the most effective life-saving therapeutic strategy for end-stage liver disease patients 1. Following the development of surgical techniques and immunosuppressive therapies like cyclosporine, graft survival remarkably improved after LT in the 1980s 2. However, the procedure is still burdened by serious postoperative complications 3. Initial studies predominantly addressed challenges from acute rejection and perioperative management 4. In recent years, the research focus has shifted toward advancements in machine perfusion, innovative graft regeneration, novel biomarkers indicating poor prognosis, and long-term complications including post-transplant metabolic disorders, cardiovascular disease, and chronic graft rejection 5. LT quality is widely affected by multiple factors arising from the donor, the recipient, the graft, and their interactions 6. Following the conundrum that emerged from increased LT demand on one side to limited organ supply on the other, more marginal grafts (such as steatotic, elderly, donation-after-circulatory-death, split grafts, etc) were introduced into clinical practice, increasing the availability of LT, but at the same time the rate of complications and early mortality in some settings 7. Frailty in recipients is strongly associated with poor post-transplant prognosis 8. Machine perfusion now offers prolonged organ preservation and even the opportunity to improve graft quality under the use of specific devices 9. Given the complexity of factors affecting LT prognosis, a better understanding of key mechanisms and molecules in these pathological processes might provide interventional targets to improve recipients' prognoses.

Hepatic ischemia-reperfusion injury (IRI) is defined as a series of pathological insults caused by hypoxia and re-oxidation processes corresponding to interruption and restoration of blood flow, respectively, which commonly occur during liver resection, transplantation, and trauma 10. IRI is a dynamic process where grafts suffer sequential injuries from warm and cold ischemia to re-perfusion considered a core mechanism of organ damage in the LT scenario 11. Of the two types of ischemia, warm ischemia in graft donation after circulatory death typically begins from the interruption of organ blood supply and lasts until perfusion is initiated, when there is no immediate blood flow restoration after asystole 12. Cold ischemia usually starts with a cooled organ flush with the hypothermic solution and ends with organ removal from a cold preservation solution for implantation. Accordingly, reperfusion injuries often occur during the re-establishment of portal vein, hepatic artery, and bile duct fluxes 13. The severity and mechanisms for reperfusion injury vary by exposure time. Reperfusion can be classified into early and late stages which correspond to shorter (within 2 h) and longer (between 6 and 48 h) times after revascularization 14, 15. Some detrimental factors, in particular graft steatosis, exacerbate post-transplant IRI 16. However, ischemic preconditioning (IPC) can alleviate the IRI process in LT patients 17. Hence, hepatic IRI is a dynamic process with variations in pathological features, sensitive pathways, and molecules in different stages 18. Accurate investigations examining mechanisms of organ injuries in specific IRI stages may help provide potential targets for graft rehabilitation during peri-transplant settings.

Due to ethical reasons, graft biopsy cannot be performed routinely at fixed time points during the whole ischemia and reperfusion process, nor can it be performed for the duration of follow-up in clinical LT studies. Furthermore, the limited tissue amounts available in these studies cannot be guaranteed to achieve research purposes. Therefore, scholars prefer to use rodents because of their significant genetic and physiological similarities to humans, especially regarding liver function and response to IRI. Rodent models are favored for their cost-effectiveness, standardization, and the availability of extensive genetic tools and reagents, making them a valuable resource for studying the fundamental mechanisms in LT and identifying key molecules/pathways relevant to human conditions 19. Animal IRI and LT models are critical tools for understanding the mechanisms and identifying potential therapeutic targets to mitigate graft damage and improve LT prognosis 20. The IRI models specifically simulate the IRI encountered during LT surgery, focusing on two sequential stages: interruption and restoration of hepatic arterial inflow. In contrast, rodent LT models provide a comprehensive approach to replicating the physiological complexities of liver transplantation, including IRI, graft regeneration, immune rejection, and surgical complications, such as early allograft dysfunction 21. By addressing different aspects of LT complications, these models are complementary and essential for advancing our understanding and management of LT-associated challenges. Further in-depth studies of transgenic livers 22, 23 can also provide effective approaches to explore the molecular mechanism for key findings in clinical LT settings. Researchers prefer to use rodents to construct IRI and LT models. However, variations may be observed in artificial settings of ischemia/reperfusion time, interruption area, graft quality, thermal condition, maneuver/pharmacologic interventions, and other conditions 24.

Omics technology provides systematic knowledge based on the quantification of whole-profile biological molecules in organisms to present the physiological/pathological process based on DNA, RNA, protein, and metabolite levels 25. Following technological improvement and algorithmic development, decreased unit price provides additional rationale for integrative omics studies to better decode potential mechanisms underlying complex phenotypes with mutual validations on systematic biological perspectives 26. Advanced single-cell RNA sequencing (scRNA-seq) has emerged to provide expression data at individual cell scales in higher resolution to explore intercellular heterogeneity and connectivity 27. Spatial omics studies depict molecular profiling in cellular/subcellular scales *in situ*, distinguishing their spatial localization, thereby offering insights for quantification on cellular organizations, interactions, and identification of functional domains in biological organisms 28. Accordingly, multiple omics technologies have been widely used in LT and IRI models for mechanistic investigation and target exploration. Omics studies focusing on factors like organ steatosis 29-33, ischemic pre/postconditioning prevention 34-37, and acute rejection 38-40 are also involved in the above-mentioned models. Further integrative omics models driven by artificial intelligence and machine-learning-based algorithms offer novel approaches for better organ allocation, perioperative management, and prognostic prediction in LT settings 41. Collective studies have been summarized based on omics results in clinical LT cases 6, 42 but have been rarely reviewed in experimental animal models. Given the heterogeneity in study design, animal species/gender, disposal time, and detection means, findings based on omics data from rodent LT and HIRI models are worth summarizing to decipher core mechanisms which cause inferior prognosis in different scenarios.

Therefore, we have summarized the published multi-omics studies based on rodent LT and IRI models categorized by study focus. Panels of susceptible molecules in different stages are summarized separately and compared with similar studies in human cases. Potential algorithms and tools for deeper data mining are depicted. Finally, prospects and research directions deserving further omics studies in rodent models are also suggested based on important topics from clinical LT issues.

## Rodent models in LT-related research

Animal models provide a platform for mechanistic investigation, treatment development, and therapeutic effect assessment in organ transplantation. Specifically, rodent LT models are highly valued due to their cost-efficiency, rapid breeding cycles, and well-established surgical procedures 43. Researchers may choose two different strategies including LT or hepatic IRI models in rodents to imitate the fluctuations observed in various scenarios in the clinical LT process 24, 44. Rodent LT models are primarily used to study the mechanisms in LT including graft dysfunction, immune response, and impacts of other factors (like graft steatosis, aging, and immunosuppressant intake) on recipients' outcomes 44. These models are also useful in exploring the impacts of clinical conditions or treatments on graft function and transplant outcomes. By imitating specific surgical maneuvers and physiological aspects in the human LT process, researchers can gain more insights into graft acceptance and long-term viability 44. By contrast, IRI models are used to explore the effects of ischemia and subsequent reperfusion on graft tissues. These models are crucial for understanding IRI as a common complication in LT procedures, and can then be used to assess the effect of potential therapeutic interventions for alleviating surgical damage 20.

LT experiments can be performed in either rats or mice. The application of rodent type is dependent on balanced considerations such as animal/organ size, genetic background, and the variety of eligible gene-edited models. Researchers should make optimal determinations based on sample volumes, lab techniques, and research budgets 45. In the coming omics era, comparisons and mutual validations of findings across different species need to be reported to increase their reliability and applicability for further translational studies 46.

## Application of omics data in rodent liver IRI models

### Major findings from animal models

The rodent hepatic IRI model is widely utilized to investigate the mechanisms of operational liver damage as well as to evaluate the efficacy of protective strategies and therapeutic interventions in the context of liver transplantation and surgical procedures 20. Following the developments in technology and decreasing costs, an increasing number of omics studies have been conducted based on rodent hepatic IRI models. Some researchers have directly applied omics data to explore the molecular mechanisms underlying IRI per se 47-53. Other studies used the IRI model as a means to investigate the effects of key factors including steatosis 33, aging 54, or interventions like ischemic pre/postconditioning 34-37 and itaconate pre-treatment 55 on liver injury severity. Transcriptomic, proteomic, and metabolomic assays were employed or integrated into each study.

To systematically identify studies on multi-omics research in rat/mouse liver IRI models, we conducted a detailed search using databases such as PubMed and Web of Science. The search strategy employed the Boolean expression 56: ("liver ischemia-reperfusion" OR "hepatic ischemia-reperfusion") AND ("omics" OR "transcriptomics" OR "proteomics" OR "metabolomics") AND ("rat" OR "mouse" OR "rodent"). The search was restricted to articles published between 1990 and 2024, written in English, which focused on experimental studies using rodent models combined with omics technologies. To ensure comprehensiveness, additional relevant studies were identified by screening the references of selected articles.

After reviewing the full text to assess its relevance and methodological quality, we identified 15 studies that applied omics data analysis in rodent liver IRI models. Data from eligible studies were extracted and are summarized in Table 1. The IRI models can be classified based on the type and duration of ischemia and reperfusion operations. Ischemia in the liver IRI model is equivalent to the warm ischemia stage in the LT model lasting between 0.5 and 1.5 h. Reperfusion varied from 1 to 24 h. Most investigations were contributed by groups from China and the U.S. Eleven and four studies were conducted in mice and rats, respectively. All studies sampled livers at different IRI stages. Bulk/spatial transcriptomics and proteomics assays were applied. For transcriptomics, RNA-seq and microarray were employed for each of the seven studies.

Noteworthily, discrepancies in study results may arise due to variability in experimental designs, such as differences in reperfusion times (e.g., short-term vs. long-term) and donor and model characteristics (e.g., steatotic or aged livers). In addition, technical differences in omics platforms like RNA-seq versus microarray, and the use of distinct bioinformatics tools also contribute to inconsistencies. Variations in ischemia/reperfusion duration, temperature settings, and surgical techniques further expand these discrepancies. These indicators of inconsistencies were collected and are presented in Table 1.

To address these inconsistency issues, we grouped studies with similar experimental conditions by comparable reperfusion durations and extracted overlapping pathways and molecules to identify consistent findings. This integrative approach reduces variability and highlights reliable molecular targets, thus providing a more robust framework for understanding the IRI process when advancing translational applications.

#### Models to explore the IRI mechanism

We collected seven studies with a focus on IRI mechanisms based on omics approaches. Omics data were collected primarily focusing on post-reperfusion liver transcriptomic variations vs. baseline. Studies were categorized into long-term and short-term reperfusion groups with 6 h as threshold.

**Figure 1:**
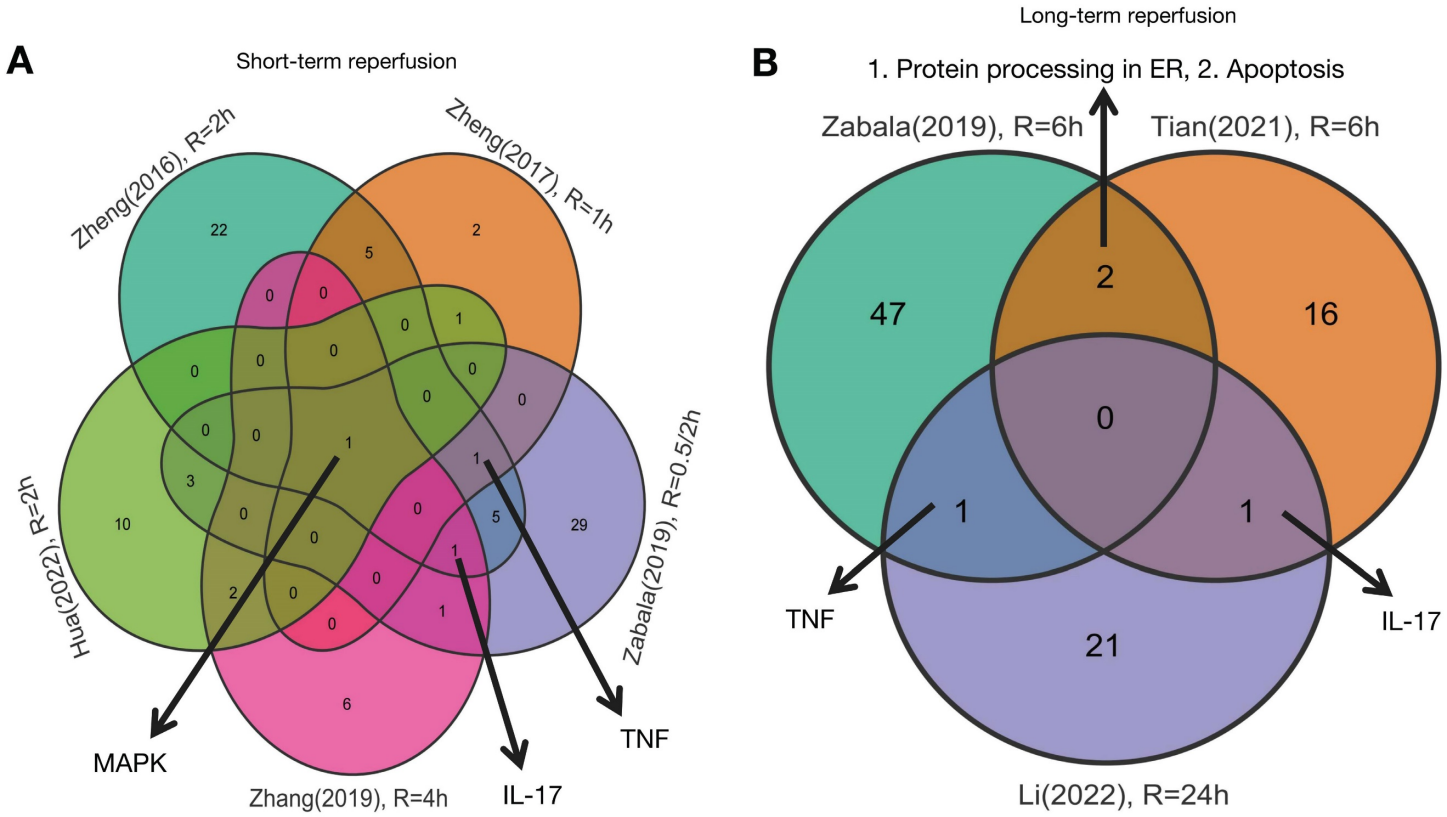
Venn plot of susceptive pathways in livers by reperfusion period. A.Venn plot of susceptive pathways in livers after short-term reperfusion; B. Venn plot of susceptive pathways in livers after long-term reperfusion. Short-term reperfusion was defined to be less than 6 hours; Long-term reperfusion was defined as equal to or more than 6 hours.

**Figure 2:**
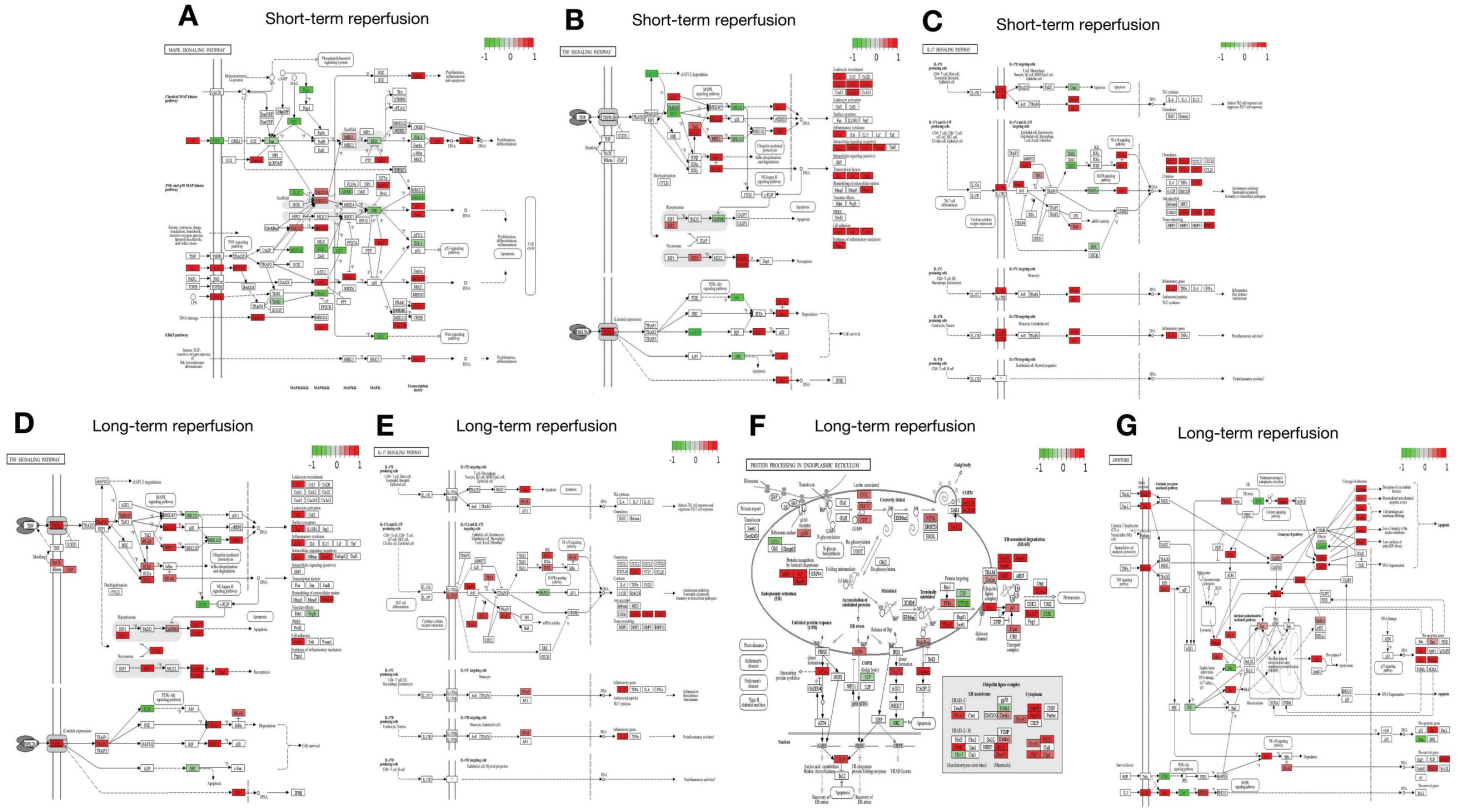
Details of signaling pathway based on DEGs from livers after reperfusion. A. Details of MAPK signaling pathway based on DEGs from livers after short-term reperfusion; B. Details of TNF signaling pathway based on DEGs from livers after short-term reperfusion; C. Details of IL-17 signaling pathway based on DEGs from livers after short-term reperfusion; D. Details of TNF signaling pathway based on DEGs from livers after long-term reperfusion; E. Details of IL-17 signaling pathway based on DEGs from livers after long-term reperfusion; F. Details of protein processing in endoplasmic reticulum pathway based on DEGs from livers after long-term reperfusion; G. Details of apoptosis pathway based on DEGs from livers after long-term reperfusion. Short-term reperfusion was defined to be less than 6 hours; Long-term reperfusion was defined as equal to or more than 6 hours. Details of DEGs of interest were visualized in the PATHVIEW website (https://pathview.uncc.edu/). Abbreviations: DEGs, Differentially Expressed Genes.

**Figure 3:**
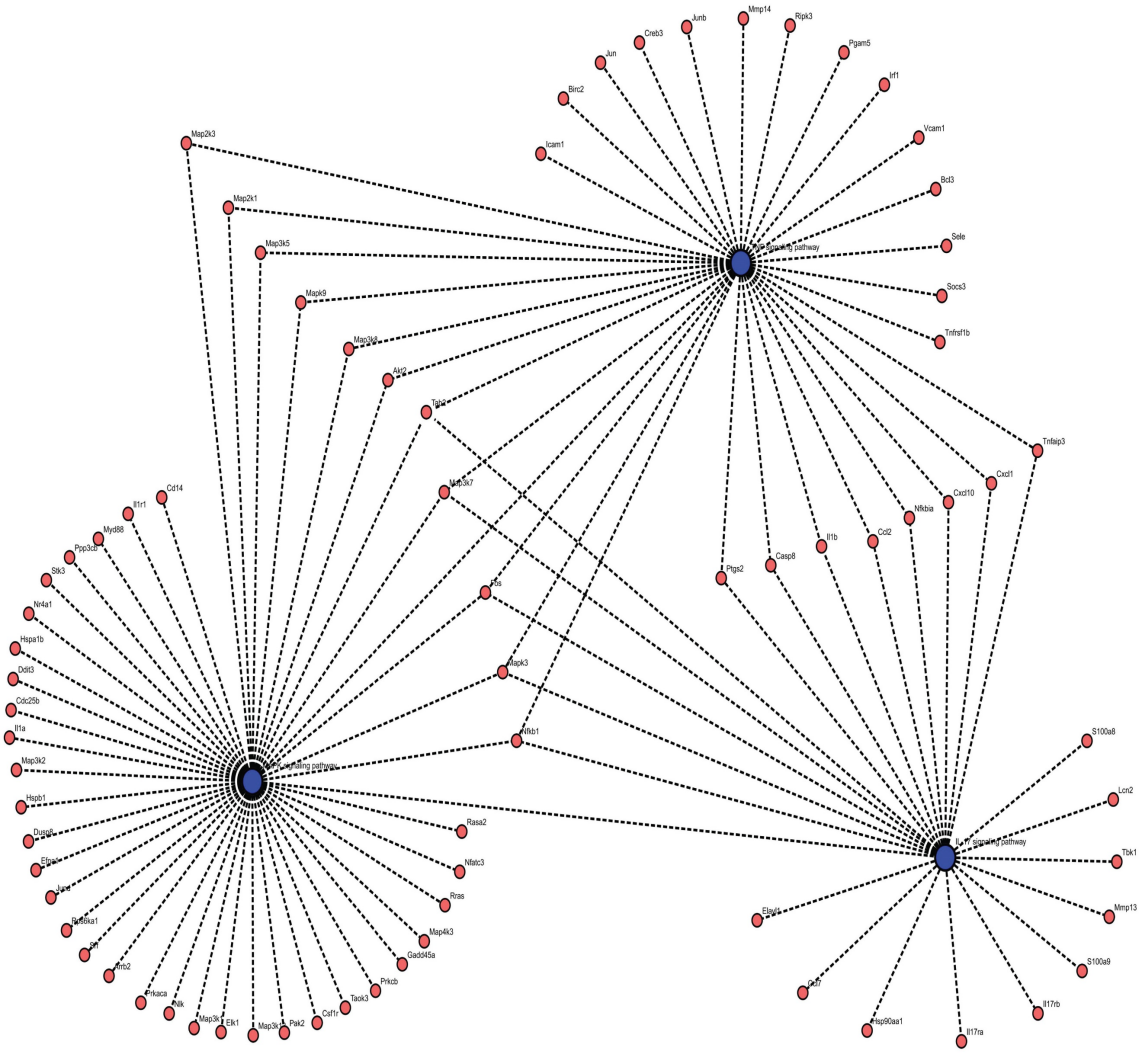
Interactions across MAPK, TNF, and IL-17 signaling pathways enriched by DEGs in livers after short-term reperfusion. Abbreviations: DEGs, Differentially Expressed Genes.

As shown in Figures 1 and 2, five studies investigated transcriptomic variation in livers with short-term reperfusion (<6 h) 34,35,48,51,52. Significant perturbations in the mitogen-activated protein kinase (MAPK) signaling pathway were observed across all studies. Significant variations in tumor necrosis factor (TNF) and interleukin (IL)-17 signaling pathways were also observed in three selected manuscripts. Based on a re-analysis of differentially expressed genes in enrolled studies 48, 52 via Kyoto Encyclopedia of Genes and Genomes pathway enrichment analysis 57, the trends for key gene variations in the above-mentioned pathways are visually presented using the PATHVIEW website (https://pathview.uncc.edu/). These pathways are closely interconnected by sharing five core genes including *Tab2*, *Map3k7*, *Fos*, *Mapk*, and *Nfkb1* (Figure 3).

Three studies explored liver transcriptomics before and after long-term reperfusion [36,47,52; Figure 1B]. As shown in Figure 2, significant alterations in TNF and IL-17 signaling pathways were observed in two of the studies. Additionally, pathways involving protein processing in the endoplasmic reticulum (ER) and apoptosis were also found dysregulated during long-term reperfusion phases.

The MAPK signaling pathway plays a pivotal role in mediating liver IRI 58. Upon reperfusion, various MAPK cascades, including extracellular signal-regulated kinase (ERK), c-Jun N-terminal kinase (JNK), and p38 are activated, triggering a range of cellular responses such as inflammation, apoptosis, and cell survival 59. This activation is crucial in the early phases of hepatic IRI, as it mediates both inflammatory responses and oxidative stress. In particular, the p38 MAPK pathway has been shown to contribute significantly to liver injury by enhancing the production of pro-inflammatory cytokines and promoting hepatocyte apoptosis 60. Additionally, the JNK pathway is involved in mitochondrial dysfunction and further amplifies oxidative stress, exacerbating liver damage during reperfusion 61. Overall, MAPK signaling pathways are integral to the inflammatory and cellular stress responses that contribute to liver IRI. Based on the integration of omics results from the enrolled literature, MAPK pathways are critically dysregulated in the early reperfusion phase. Targeting these key genes may provide therapeutic opportunities to mitigate liver damage, reduce inflammation, and improve outcomes in patients undergoing LT or other IRI-associated procedures.

The TNF signaling pathway plays a critical role in liver IRI by initiating and amplifying inflammatory and apoptotic responses 62. During the reperfusion phase, TNF-α is produced by Kupffer cells and rapidly increases in the liver 63. TNF-α binds to its receptors TNFR1 and TNFR2, triggering downstream signaling cascades that promote the expression of inflammatory mediators, including IL-6 and other cytokines and chemokines. Increased inflammatory cytokines cause the recruitment of neutrophils and macrophages, exacerbating oxidative stress and tissue damage 64. In addition, TNF signaling also causes hepatocyte apoptosis through mitochondrial dysfunction and caspase activation, leading to subsequent liver injury 65. Dysregulation of key genes in the TNF signaling pathway was observed throughout the whole reperfusion phase in IRI so key genes in the TNF pathway may be potential therapeutic targets to mitigate the IRI process by reducing inflammation and apoptosis.

Finally, the IL-17 signaling pathway impacts IRI by driving pro-inflammatory responses to exacerbate tissue damage 66, 67. IL-17 recruits and activates neutrophils/macrophages, promoting cytokine/chemokine storms and inflammation at the injury site, contributing to immune-mediated pathology 68.

The MAPK, TNF, and IL-17 signaling pathways all engage in complex interactions with synergistic amplifications of inflammatory responses in the IRI process. MAPK activation including ERK, JNK, and p38, triggers more production of pro-inflammatory cytokines including TNF-α and IL-17 to aggravate IRI severity 69. Meanwhile, TNF-α also activates the MAPK cascades, establishing a feed-forward loop to sustain inflammation and cell death 70. IL-17 intensifies the IRI process by promoting neutrophil recruitment and oxidative stress under stimulated TNF-α and MAPK expression 71. Crosstalk across the above-mentioned pathways leads to increased vascular permeability, apoptosis, and organ dysfunction, offering therapeutic potential to mitigate IRI 72.

Intriguingly, genes related to apoptosis and protein processing in ER pathways are also dysregulated in livers after prolonged reperfusion. Apoptosis causes irreversible damage by regulating programmed cell death through caspase activation and mitochondrial dysfunction 73. Protein processing in the ER refers to events that occur in the proper folding, modification, and transportation of newly synthesized proteins. Dysfunctions in this pathway can lead to the accumulation of misfolded proteins associated with IRI 74. These changes highlight the trend of exacerbated ER stress and apoptotic signals in livers after extended reperfusion.

Concerning post-transcriptional regulation of gene expression, Zheng *et al.* 48 performed microRNA transcriptomic profiling of the reperfused liver and found 69 differentially expressed microRNAs between the reperfusion samples and the sham surgery control group. These differentially expressed microRNAs mainly participated in immune and inflammatory responses during reperfusion. Specifically, microRNA (miR)-125b-5p and miR-501-3p were downregulated during reperfusion, leading to the upregulation of their target genes *Myd88*, *c-Fos,* and *A20*, which in turn activated the Toll-like receptor signaling pathway, triggering immune and inflammatory responses.

#### Application of advanced technology in IRI models

Advanced omics technologies including scRNA-seq and spatial omics have significantly enhanced our understanding of cellular and functional locations for mechanisms underlying disease progression 75. However, applications of these technologies in animal IRI models remain largely underexplored. Xin *et al.* utilized spatial transcriptomics to analyze zone-dependent hepatic IRI in a mouse model, revealing that the pericentral zone is most susceptible to IRI 75. However, further analysis of differentially expressed genes and pathways active in specific locations was not analyzed in this study.

#### Models to Investigate Marginal Grafts on IRI

Marginal liver graft refers to a series of defects in donor/grafts, which make the organ sub-optimal for transplantation or more susceptible to post-operative complications. In particular, graft steatosis and donor aging are two major defects in various types of the marginal liver 76 with a detrimental impact on the organs and recipients' survival 77. IRI plays a key role in the connection between marginal grafts and poor prognosis 76. Omics data are crucial for identifying the molecular mechanisms of amplified IRI in marginal grafts, which may provide potential biomarkers and therapeutic targets to improve post-transplant outcomes.

For steatosis, our previous study found graft macrosteatosis was associated with more than 2.8-fold higher graft mortality in Chinese people who underwent LT 78. Graft steatosis exacerbates IRI severity by enhancing ferroptosis, oxidative stress, and inflammatory cytokine release which leads to more severe mitochondrial dysfunction, cellular apoptosis, and graft injury 79-81. As shown in Table 1, Tiriveedhi *et al.* employed proteomic assays to explore the variation of protein profiles in steatotic livers from obese Zucker rats during the ischemia/reperfusion (IR) process 33. Significant down-regulation of molecular chaperones was observed in steatotic livers after IR treatment. Chaperones present therapeutic potential as targets to improve the viability of steatotic allografts for further translational study.

As another key feature of marginal livers, donor aging also exerts adverse impacts on LT prognosis. Based on a meta-analysis of published data, we found a prominent decrease in 1-year graft/recipient survival in cases utilizing aged grafts 82. Aging livers exacerbate the IRI process because of the reduced regenerative capabilities and impaired mitochondrial function, which has been linked to a poor prognosis after LT 83. Based on microarray data from aging mouse livers experiencing IR treatment, Huber *et al.* observed that aging was associated with a significant decrease in proteasome expression, especially for 26S proteasome non-ATPase regulatory subunit 4, which could lead to defective nuclear factor kappa B activation during hepatic IRI 54. These results underscore the impact of age-related molecular alterations on graft quality.

Despite the importance of marginal grafts in the IRI process, the results of omics studies focusing on IRI in marginal grafts are still insufficient to provide robust conclusions after meta-analysis that can provide new insights to improve organ preservation.

#### Models to investigate operational factors on IRI severity

Given the central role of IRI in liver surgical damage, preconditioning for IRI mitigation is crucial for preserving liver function, minimizing complications, and improving overall patient recovery 84. Numerous studies have explored strategies to mitigate IRI, including operative ischemic pre/postconditioning, pharmacological interventions, or key gene editing 79. Omics data provide valuable insights into the molecular mechanisms underlying these interventions and facilitate the identification of specific targets to improve IRI management.

Ischemic conditioning (IC), including ischemic postconditioning (IPO) and ischemic preconditioning (IPC), is an available strategy performed during or before ischemic events to enhance tissue resistance to IRI 14. IC is widely utilized in surgical procedures including liver resection and LT to reduce IRI 85. The mechanisms for IC-associated IRI alleviation are mainly involved in antioxidant activation, inflammatory modulation, mitochondrial protection, and cell apoptosis reduction can promote liver repair and regeneration 79.

As shown in Table 1, there were four studies depicting the transcriptomic variations in rodent livers receiving IC before IR treatment, including two studies on IPC 34,36, one study on IPO 35, and one study applying both treatments for IRI alleviation 37. Two studies 23,25 investigated the effect of IPC and IPO on short-term reperfusion, which found that IC regulated the MAPK, IL-17, and TNF signaling pathways, as was also reported in pure IRI omics studies (Figure 2). Our prior RNA-seq data on livers by IPC and long-term reperfusion (6 h) showed no significant alterations in the abovementioned IRI-related pathways 36, suggesting that IC might exert more protective effects on short-term reperfusion. However, more data are needed to confirm this speculation. A Danish study compared the combined protective effects of IPC and IPO on IRI alleviation 37 and reported that IC activated the regulatory networks in cellular homeostasis and proliferation to resist IRI. However, combined IPC/IPO treatment did not confer additional IRI protection based on gene profiling levels. Additionally, Hua *et al.* explored the significance of long non-coding RNA profiles in IC-mediated mitigation of IRI 55 and found that they may contribute to liver protection during the IRI process by regulating cellular stress responses and modulating the MAPK pathway. Our data suggest that the circular RNA circRNA_017753 may regulate Jade1 expression by interaction with miR-218-5p, miR-7002-3p, and miR-7008-3p to exert IPC-mediated liver protection by modulating apoptosis, stress responses, and cell regeneration in the IRI process 36.

Pharmacological interventions also play a significant role in mitigating ischemia-reperfusion injury by targeting various pathological mechanisms, including oxidative stress, inflammation, and apoptosis. Antioxidants such as N-acetylcysteine reduce oxidative damage by scavenging reactive oxygen species, while anti-inflammatory agents like dexamethasone inhibit the inflammatory cascade that exacerbates tissue injury 86. Additionally, IC mimetics such as adenosine and mitochondrial protectants like cyclosporine A have shown potential in preserving mitochondrial function and preventing cell death 87. These interventions aim to protect cellular integrity, enhance tissue recovery, and ultimately improve clinical outcomes. Xu *et al.* revealed the itaconate intervention mitigated hepatic IRI by targeting genes in the MAPK and IL-17 signaling pathways 34. Meanwhile, itaconate administration stimulated tissue repair and metabolic regulation by activating stem cell pluripotency and insulin resistance. Omics data help in evaluating the effects of drug interventions on IRI mitigation.

Gene editing also holds promise for alleviating IRI in grafts by modifying the expression of key genes involved in oxidative stress, inflammation, and apoptosis. Techniques like CRISPR/Cas9 targeting precise genes may be used to examine their ability to modulate IRI 88. Omics data can validate the importance of these regulations by confirming the susceptive pathways involved in transgenic grafts through an unsupervised approach 89.

Macrophage migration inhibitory factor (MIF) can activate the MAPK signaling pathway including JNK and P38 playing central roles in cellular stress responses and inflammatory processes 90. Chen *et al.* revealed that the absence of MIF suppressed the apoptosis signal-regulating kinase 1-JNK/P38 axis on the MAPK signaling pathway based on transcriptomics data, indicating a crucial role for MIF in inflammation and apoptosis during IRI 91.

Toll interacting protein (Tollip) interacts with various components of the Toll-like receptor signaling cascade to regulate inflammatory signal transduction 92. Yan *et al.* found liver-specific Tollip knockout significantly alleviated liver IRI by inhibiting MAPK signaling and subsequent inflammation, apoptosis, and tissue injury in the IRI process based on omics data 53. Furthermore, gp78 is an E3 ubiquitin ligase enzyme found on the ER membrane. It is involved in cellular metabolism and inflammation by mediating the ubiquitination of various substrates in cholesterol metabolism 93. Our prior study found differentially expressed molecules in livers with over-expressed Gp78 mainly located in ferroptosis, fatty acid metabolism, oxidative stress response, and polyunsaturated fatty acid metabolic pathways based on multi-omics data from a mouse hepatic IRI model 22.

In summary, omics assays are widely utilized in rodent IRI models, offering valuable insights to uncover the IRI mechanisms at the molecular level. Meanwhile, omics data provide available approaches to assessing strategies for IRI prevention including operational procedures, pharmacological treatments, and genetic modifications.

## Comparison between rodent IRI and LT models

Both IRI and LT models in rodents reflect certain processes involved in the clinical LT process, providing valuable and reproducible models for understanding the mechanisms of LT complications and involved risk factors 45. IRI models are easy to operate and highly controllable which can imitate the key damage process in LT. They can be used to investigate the individual or combined effects of surgical or graft-specific factors to uncover the underlying pathophysiological mechanisms of organ damage including oxidative stress and inflammatory responses. Usually, the time setting for IRI is defined within 24 h, suitable for high-throughput screening of drugs for relieving short-term LT damage 24. The IRI model avoids the confounding effects of allogenic immune rejection and inconsistent surgical operation, helping to better illustrate the injury mechanisms in LT 20.

Unfortunately, IRI models cannot simulate immune rejection or long-term complications like graft fibrosis or tumor recurrence. As we know, dynamic variations are observed in some indicators of short-term duration after LT, and IRI models fail to precisely evaluate the impact of variation of donor-related risk factors (such as steatosis) on LT injury 94. In addition, extrahepatic influence cannot be assessed in IRI models. Notably, the ischemia type in the IRI model is warm ischemia without organ removal, which does not align with clinical LT settings with shorter warm ischemia *in vivo* but longer cold ischemia* ex vivo* 95. Furthermore, the IRI model cannot reflect well the natural course of LT-related damage.

By contrast, rodent LT models closely imitate clinical processes which can be used as an ideal model to evaluate immune graft rejection/tolerance, immunosuppressant efficacy, graft regeneration, post-operative organ function, tumor recurrence, and recipient survival. Rodent LT models are crucial to uncover further complex mechanisms by simulating interactions combining various factors in clinical settings 21,44. Noteworthy, donor-recipient interaction can only be simulated by the LT model.

However, rodent LT is a complex procedure with high demands on the technician's expertise. Longer recovery limits the use of the LT model in high-throughput drug screening. Additionally, immunological differences across individual donors and recipients contribute to significant variability in follow-up observations, affecting the reproducibility and reliability of results in rodent LT models 96, 97. A comparison between IRI and LT models is concisely presented in Table 3.

Therefore, future omics research should prudently consider the selection of animal models. Multi-modality data integrating omics data across animals, cells, and clinical cases will help to cross-validate data reliability and improve its translational clinical application.

## Application of omics data in rodent LT models

By contrast, few studies have applied omics approaches in rodent LT models. As shown in Table 2, six relevant studies 29-31, 98-100 were enrolled for further analysis. Of these, three studies investigated steatotic graft application in LT, two studies explored rejection after LT, and one examined the post-transplant biochemical variations in recipients. For omics assays, one study used advanced single-cell sequencing, two applied multi-omics integration, and another three explored metabolic profiling to address their key concerns.

As previously mentioned, steatotic grafts cause more complications and poor post-transplant prognosis via increased IRI, impaired regeneration, and immune responses. Based on multi-omics integration and advanced single-cell sequencing, the mechanisms underlying poor outcomes caused by steatotic grafts were explored from various perspectives. Based on sequential biopsy samples, we found post-transplant steatosis caused inferior graft prognosis via suppressed E2 promoter binding factor 1-centered hepatocyte proliferation and antioxidant dysfunction 29. Yang identified fatty acid-binding protein 4 (FABP4) as a potential therapeutic target in steatotic LT, as FABP4 inhibition could reduce oxidative stress by activating cAMP signaling pathways in post-transplant grafts 31. A subsequent single-cell RNA-seq study found that immune cell heterogeneity was present in steatotic grafts, with a differentiated trajectory of myeloid cells linked to immune-metabolic imbalance. Specific cell types, including Kupffer cells and Dendritic cells (DCs), played key pro-inflammatory roles in LT damage, and the interaction between chemokine receptor XCR1^+^ DCs and CD8^+^ T cells may exacerbate the IRI process 30.

Graft rejection is a complex immune-mediated process in which the recipient's immune system recognizes the grafts as foreign 101. The model using Lewis rats as donors and Brown Norway rats as recipients is the most common model to induce graft rejection 102. Previous metabolomics studies have shown that post-transplant serum/plasma metabolic profiling can be used to predict post-transplant rejection 98,100. Wang *et al.* explored the expression levels of liver/serum metabolic profiles after LT in rats, identifying metabolites that could characterize post-transplant metabolic patterns and provide new insights into the pathophysiology of post-transplant grafts 99. However, all referred papers published around 2010 only identified a few metabolites that lacked metabolite enrichment and failed to explore biological pathways for the underlying mechanisms.

## Current concerns and challenges

Given the key role of reperfusion injury in the LT process, the major findings regarding IRI models can be presented as: 1. Except for pure mechanistic investigations, multi-omics data have contributed significantly to advance the possibility of comprehensively assessing pre-ischemic interventions or pharmacological treatments for alleviating LT damage, as well as in validating transgenic models for reducing IRI severity; and 2. Inflammatory signaling pathways including TNF and IL-17 showed significant disruptions throughout the entire reperfusion process (Figure 2). Molecules involved in the MAPK pathway like MIF played a crucial role in blocking IRI progression 91.

Conversely, limitations of the current IRI models are also evident as follows: 1. Most studies lacked cross-species validation of the omics data with human data to enhance the therapeutic potential of findings from rodent models; 2. Key molecules cannot be located in the absence of transcriptional regulatory networks addressed in most studies, reducing their applicability in translational investigations; 3. Some studies showed low quality with the absence of biological replicates 50; and 4. Notably, scRNA-seq has never been performed in rodent IRI models, which represents a significant gap in our current understanding of key cellular populations and biomarkers involved in IRI regulation. Despite the critical importance of uncovering the cellular heterogeneity and dynamic molecular interactions that underlie the disease processes, scRNA-seq in rodent models is relatively scarce. We are now conducting and analyzing the original sc-RNA seq data from specific rodent IRI models aiming to address this gap and contribute novel insights that can advance the field and bridge its translational relevance to human studies. Omics studies in rodent LT models are relatively scarce, and their applications have not been fully explored. The need for higher technical expertise and low survival rates have prevented their wider application. Recently, concerns about omics data from the LT model have been mainly restricted to exploring the use of marginal grafts in clinical settings 29-31.

Many risk factors from the donor, recipient, surgery, and graft 6, 103 have not been addressed in omics data in the LT model. Additionally, novel technologies like spatial omics were not reported in prior rodent LT studies, limiting further the understanding of tissue organization and cellular interactions in graft biological systems.

## Future perspectives and translational implications

As mentioned before, the rodent IRI model cannot fully mimic the reperfusion injury in clinical LT settings. Damage severity and disturbed signaling pathways were significantly distinguished by reperfusion time points. Cross-validation by results from cellular experiments 104, clinical cases, or even microfluidic Liver-On-Chip 105 will enhance the reliability and translational value of omics data from rodent models. Machine learning (ML) techniques apply data dimension reduction, differential expression analysis, pattern recognition, and multi-omics data integration. ML application to the analysis of omics data from rodent IRI model will likely help to identify the damage patterns, predict hub gene clusters, and construct transcriptional regulatory networks, advancing personalized identification for IRI treatment 106.

Advanced omics techniques including scRNA-seq and spatial omics may also provide a wide and more granular perspective to precisely understand the molecular mechanisms by revealing liver cell heterogeneity, spatial distribution, dynamic variations of cellular interactions, microenvironmental dynamics, and immunoregulations in molecule profiling in rodent IRI and LT models. These approaches will uncover novel pathways and cell-cell communication networks that drive graft dysfunction or regeneration, offering opportunities for therapeutic intervention 107, 108. Meanwhile, these techniques facilitate the identification of specific cell types, pathways, and potential targets, offering a theoretical platform to develop novel therapies to enhance IRI treatment 109. Song *et al.* employed scRNA-seq to analyze gene expression at the individual cell level in nonalcoholic steatohepatitis models, identifying key cell populations and the involved signaling pathways. ML algorithms can be used to screen the core genes in nonalcoholic steatohepatitis progression, providing targets for therapeutic intervention 110. Advanced omics study with ML algorithms is needed to be implemented in further well-designed rodent models. Moreover, integrating multi-omics datasets requires close collaboration between computational biologists and experimental researchers to ensure robust validation and reproducibility. Such efforts are critical for translating findings from rodent models to human liver transplantation settings, ultimately bridging preclinical insights with clinical applications 6, 111.

Currently, rodent LT models are mainly designed to improve transplant outcomes by modifying donor livers (Table 2). However, recipient features like frailty and weakness have gained more attention due to their negative impacts on transplant outcomes 112-114. Our prior study also found that recipient frailty characterized mainly by myosteatosis and sarcopenia exerted adverse impacts on transplant outcomes 115. However, the selection of indicators to provide an effective and simple description of frailty is controversial 116. Long-term immunosuppressant medication is associated with an increased risk of new-onset metabolic syndrome, diabetes, cardiovascular complications, malignancies, and renal dysfunction, significantly affecting the recipients' prognosis 117,118. A reliable recipient-specific rodent model with integrative multi-omics data on sequential sampling within the whole LT process would help to expand the application of the rodent model in further LT research.

Insights gained from multi-omics data in rodent IRI and LT models have significant practical implications for clinicians and researchers. The identification of key mechanisms including perturbations of MAPK, TNF, and IL-17 signaling pathways, provides therapeutic targets to mitigate liver damage and improve transplant outcomes.

Inspired by multi-omics data, interventions in the p38 MAPK pathway, which promotes IRI through pro-inflammatory cytokine production and hepatocyte apoptosis, could reduce inflammation and improve transplant outcomes 58. Additionally, the role of TNF signaling in amplifying inflammation and apoptotic responses during the IRI process highlights the therapeutic potential of TNF inhibitors (like alpinetin) in mitigating liver damage in clinical settings 119, 120.

Furthermore, the understanding of molecular mechanisms underlying LT using marginal steatotic or aging grafts can guide the development of strategies to improve graft quality and recipient outcomes. FABP4 was identified as a crucial factor for poor LT prognosis using steatotic grafts. Subsequent application of FABP4 inhibitor effectively alleviating IRI in steatotic mouse livers is validation for omics data 31. These trials facilitate the translation of omics findings into practical advancements for LT settings.

## Conclusion

In conclusion, this review summarized the application of multi-omics data in rodent IRI and LT models, integrating the key pathways and critical molecules associated with reperfusion injury and prognosis in clinical settings. Technological developments and cost reduction are markedly expanding the application of high-throughput data in rodent LT models. In the coming years, integration of advanced omics assays with rodent models based on LT research hotspots will likely provide new valuable insights for an in-depth understanding of mechanisms underlying LT injury and therapeutic potentials.

## Figures and Tables

**Table 1:** Main features of studies with omics application in rodent ischemia and reperfusion injury model

Author, country, publication year (ref.)	Species/Gender /age or weight /case vs. control	IRI model	Intervention/ graft traits	Sampling/ time	Assays/ platform	Omics	Comparison	Statistics	Major findings	Validation	Database number
Huber, USA, 2009 54	C57BL6 mice/male/4wks or 1y/3 vs. 3	WI (left+middle lobe) WI=60min, WI=90min, WI=90min, R=1h	None/aging	Liver/at the end of each treatment	MicroArray/ Affymetrix Mouse Genome MOE 430 2.0 GeneChip	mRNA transcriptomics	Case vs. shame	FDR adjusted multiple testing correction	Genes on protein ubiquitination and the proteasome pathway were significantly downregulated in old mice during I/R. PSMD4 with recognition and recruitment of ubiquitinated substrates to the proteasome for degradation was found to be reduced in old mice.	Tissue WB Cellular experiment	GSE10657
Tiriveedhi, USA, 2012 33	Zucker rats/male/8-10 wks/3 vs.3	70% WI (left+middle lobe) WI=45min, R=1h	None/steatotic	liver/at the end of each treatment	DE combined HPLC/MS	Prote- omics	Steatotic vs. non-steatotic after I/R treatment	Batched t-test	Significant changes were observed in 105 proteins after warm I/R injury in steatotic livers. Molecular chaperones including Hyou1, Cabp1, Calr, Hsp60, Hsp90B1, and Pdia3 were down-regulated after I/R in the steatotic liver, and only one chaperonin (Hspd1) was up-regulated. DE proteins in the steatotic liver can be categorized into four functional groups: molecular chaperones/ER stress, oxidative stress, metabolism, and cell structure. Down-regulated chaperones lead to increased ER stress, causing apoptosis and necrosis in steatotic livers.	None	None
Knudsen, Denmark, 2012^37^	Wistar rats/male/300-350g/10 vs. 8	100% WI, WI=30min, R=30min	IPC+IPO/none	Liver/at the end of each treatment	MicroArray/ Affymetrix Rat Exon 1.0 ST array	mRNA transcriptomics	I/R vs. shame	Significance analysis of microarrays	1. IPC and IPO exert protective effects by regulating the same gene sets, suggesting these two treatment approaches trigger similar molecular mechanisms to mitigate I/R injury.	Tissue qRT-PCR	GSE24430
		IPC^a^, WI=30min, R=30min					1. IPC vs. shame		2. 172 genes were identified in IPC and IPO groups, involved in cellular growth, proliferation, and maintenance of cellular homeostasis.		
		WI=30min, IPO^b^, R=30min 4. IPC, WI=30min, IPO, R=30min					2. IPO vs. shame 3.IPC+IPO vs. shame		3. IPC and IPO significantly affected the expression of immediate early genes, typically as TF, respond to cellular stress, and regulate cell proliferation and differentiation, including Btg2, Egr1, Myc, Fos, Jun, Atf3.		
Zheng, China, 2016^48^	C57BL6 mice/male/ 8-10 wks/ 6 vs. 6	70% WI (left+middle lobe) WI=90min WI=90min, R=2h	None/normal	Liver/at the end of each treatment	RNA-seq/ Illumina HiSeq2000	mRNA/ miRNA transcriptomics	I/R vs. shame I vs. shame I/R vs. I	edgeR package	1. In the ischemic phase, injury mainly affects mitochondrial function, nutrient consumption, and metabolic processes 2. In the reperfusion phase, injury caused severe tissue inflammation and innate immune response 3. Downregulation of miR-125b-5p and miR-501-3p in the reperfusion phase activated the Toll-like receptor signaling pathway and inflammatory response.	Tissue qRT-PCR	GSE72315
Zheng, China, 2017^51^	C57BL6 mice/male /1y/3 vs. 3	WI (left+middle lobe) WI=90min, R=1h WI=90min	None/aging	Liver/at the end of each treatment	MicroArray/ Affymetrix Mouse Genome MOE 430 2.0 Gene Chip	mRNA transcriptomics	I/R vs. I	Limma package	TNF signaling, Malaria Influenza A, and MAPK signaling pathways were detected as the top pathway to be associated with IRI Hub genes for IRI were focused on FOS, CCL2, CXCL1, JUN, IL6, and DUSP1, and all were upregulated.	None	GSE10657
Zabala, USA, 2019^52^	Wistar Rats/male/ 5-6 wks/ 3 vs.3	WI (left+middle lobe)	None/normal	Liver/at the end of each treatment	MicroArray/Affymetrix Rat Transcriptome 1.0	mRNA transcriptomics	R vs. non-R	Limma package	1. In the early stages of reperfusion, particularly at 0.5-hour, significant increase in expression of immediate-early genes like c-Fos, c-Jun, Atf3, and Egr1 in the reperfused lobe, indicating a tissue-autonomous response to reperfusion.	Tissues WB and qRT-PCR	GSE117915
		1. WI=30min, R=0min					R vs. shame		2. At the 2- and 6-hour reperfusion, a significant overlap in gene expression changes between reperfused and non-ischemic lobes, suggesting non-autonomous responses triggered by systemic circulatory factors and hemodynamic changes.		
		2.WI=30min, R=0.5h					non-R vs. shame		3. Multiple potential upstream regulators in the reperfusion phase, including cytokines (PDGF BB, IL1B, TNF, IGF1), immediate-early genes (ATF3, EGR1, FOS, JUN), and factors related to extracellular matrix and cytoskeletal remodeling (TP53, PDGF, TGFB1).		
		3. WI=30min, R=2h							4. At the 6-hour reperfusion time point, upstream regulators associated with normal liver metabolic functions, such as HNF4A, RICTOR, and MYC indicate a trend toward the restoration of normal liver metabolic functions during the recovery process.		
		4. WI=30min, R=6h							5. FGF21 may play a role in protective responses following IR injury.		
Zhang, China, 2019^35^	C57BL6 mice/male/9 wks/3 vs.3	70% WI WI=1h, R=4h WI=1h, (5 sec I/R cycles)*3, R=4h	IPO/none	Liver/at the end of each treatment	RNA-seq/Illumina HiSeq 4000	mRNA transcriptomics	IPO+I/R vs. I/R I/R vs. shame	Ballgown package	1.2416 DEGs were identified between the normal, I/R, and IR+IPO groups. 2. KEGG pathways included the MAPK pathway, IL-17 signaling, stem cell pluripotency, and insulin resistance pathway associated with IPO. 3. Twelve DEGs were validated by qRT-PCR, of which 11 genes (including Cyr61, Atf3, Nr4a1, Gdf15, Osgin1, Egr1, Epha2, Dusp1, Dusp6, Gadd45a, and Gadd45b) showing significantly increased expression after IPO.	Tissue qRT-PCR	GSE117066
Yan, China, 2019^53^	C57BL6 mice/male/ 8-14wks/ 4 vs. 3	70% WI (left+middle lobe) WI=1h, R=6h	None/Tollip KO	Liver/at the end of each treatment	RNA-seq/ BGISEQ 500	mRNA transcriptomics	Tollip KO vs. WT after IR treatment	DeSeq2 package	1. GSEA found Tollip absence inhibited biological processes related to adhesion, migration, infiltration, and inflammatory activation. 2. Genes associated with cell death were downregulated in Tollip KO mice. 3. KEGG found PI3K-AKT signaling pathway, cytokine-cytokine receptor interaction, and MAPK pathways were the top three molecular events contributing to Tollip function."	Tissue WB, TUNEL	NA
Tian, China, 2021^36^	C57BL6 mice/male/ 18-22g/ 3 vs.3	70% WI WI=1h, R=6h IPC (WI=10 min, R=10min), WI=1h, R=4h	IPC/none	Liver/at the end of each treatment	MicroArray/Agilent array	mRNA transcriptomics	I/R vs. shame	Limma package	In the IRI model, 39 circRNAs and 432 mRNAs were upregulated, 38 circRNAs and 254 mRNAs were downregulated. After IPC intervention, 43 circRNAs and 64 mRNAs were upregulated, 7 circRNAs and 31 mRNAs were downregulated. circRNA_017753 was identified as a target for IPC protective signaling.	tissue qRT-PCR	GSE164367
						cirRNA transcriptomics	2. IPC vs. shame		3.Three axes (circRNA_017753-miR-218-5p-Jade1, circRNA_017753-miR-7002-3p-Jade1, circRNA_017753-miR-7008-3p-Jade1) might play important roles in IPC-related IRI protection.		
Xu, China, 2021^55^	C57BL6 mice/male/ 8wks/ 5 vs. 5	70% WI (left+middle lobe) 1. OI inject, WI=90min, R=6h	IPC/none	Liver/at the end of each treatment	MicroArray/Illumina, TruSeq PE Cluster Kit V4	mRNA/ lncRNA transcriptomics	OI-con+I/R vs. OI-con+Sham	DeSeq2 package	1. Itaconate can alleviate IRI in mouse livers. OI decreased the release of inflammatory cytokines and mitigated liver tissue damage.	tissue qRT-PCR	PRJNA 702236
		2. OI-con inject, WI=90min, R=6h 3. OI inject, shame 4. OI-con inject, shame					OI+I/R vs OI-con+IR OI+I/R vs OI+shame		2. 138 lncRNAs and 156 mRNAs were differentially expressed in the OI+I/R group. 14 pathways were significantly enriched in both I/R and OI+I/R groups, including TNF, chemokine, malaria, Salmonella infection, NOD-like receptor, rheumatoid arthritis, PI3K-Akt, and Toll-like receptor pathway.		
							OI+shame vs OI-con+shame		3. PPIN found eight genes associated with OI-related IR alleviation, including IL6, IL1B, PTGS2, MMP13, CCL3, CCL4, OSM, and IL1F9.		
Li, China, 2022^47^	SD rats/ male/ 10 wks/ 10 vs. 10	70% WI (left+middle lobe) WI=90min, R=24h	None/normal	Liver/at the end of each treatment	RNA-seq/PE150, Novaseq 6000	mRNA/ miRNA transcriptomics	I/R vs. control (untreated)	DeSeq2, machine learning algorithm	1. Six genes (Krt14, Upk3b, Krt7, Cdh3, Msln, and Gpc3) were defined as the key genes on IRI. 2. DEGs were mainly enriched in IL-17 signaling pathways, lipid and atherosclerosis, and retinol metabolism. 3. lncRNA-miRNA-mRNA network for IRI related key genes: LOC120102987-rno-miRNA-331-3P-Cdh3, LOC1201029870-rnomiRNA-128-5p-UPK3B, and LOC120094223-rno-miRNA-92b-5p-KRT7.	Tissue qRT-PCR	None
Chen, China, 2022^91^	C57BL6 mice/male/8-10wks/3 vs. 3	70% WI (left+middle lobe)	None/MIF OE	Liver/at the end of each treatment	RNA-seq/HiSeqTM 2,500 or Illumina HiSeq X	mRNA transcriptomics	MIF KO vs. WT after IR treatment	DeSeq2 package	1. KEGG/GSEA found the MAPK signaling pathway was downregulated for MIF-mediated IRI. 2. SK1 (MAP3K5) was downregulated in MIF-KO-mediated IRI.	Tissue WB	GSE212508
		1. WI=1h, R=6h							3. MIF activates SK1-JNK/P38 signaling pathway to cause IRI.		
Hua, China, 2022^34^	C57BL6 mice/ male/ 8-10 wks/ 6 vs. 6	70% WI (left+middle lobe)	IPC/none	Liver/at the end of each treatment	MicroArray/Arraystar Mouse LncRNA Microarray V3.0	mRNA/ lncRNA/ transcriptomics	1.IPC+I/R vs. I/R	One-way ANOVA using Dunnett t-test	167 DE lncRNAs and 108 mRNAs were identified in IPC+I/R groups. KEGG analysis indicated DEGs were enriched in protein processing in the endoplasmic reticulum, antigen processing and presentation, and fructose and mannose metabolism.	Tissue qRT-PCR	GSE192977
		IPC (I=10min, R=10min), WI=75min, R=2h WI=75min, R=2h					2.I/R vs. shame		A weighted co-expression network was constructed, and 7 DEGs (Hspa1ab, Chka, Clec2h, Mvd, Adra1a, AK085737, and AK088966) were identified and validated by qRT-PCR. Key regulatory axes like AK088966/mmu-miR-6349/Chka and Adra1a/mmu-miR-7657/Mvd have been involved in IPC-related IR alleviation.		
Xin, China, 2023^49^	C57BL6 mice/male/20-25g/NA	70% WI (left+middle lobe)	Celastrol treat/none	Liver/at the end of each treatment	RNA-seq/Illumina NovaSeq 6000	Spatial transcriptomics	1. Same zone in I/R and shame group	B-H adjust p-values and fold changes	Samples are divided into three zones: Zone 1: Near portal vein; Zone 2: Mid-region between zone 1 and 3; Zone 3: Near central vein. 1. Celastrol pre-treatment reduces IRI via modulations on inflammatory and hypoxia pathways in Zone	Tissue qRT-PCR and IHC	GSE217936
		1. WI=1h, R=6h		Regions in different zones were assayed by RNA sequence			Same zone in I/R and celastrol -treated Group		2. After I/R injury in zone 3, M1/M2 ratios increased. Celastrol restored M1/M2 balance and reduced inflammation.		
		2. Celastrol inject one week before, WI=1h, R=6h					Comparisons between different zones in I/R or celastrol- treated groups.		3. Celastrol may reduce IRI via ischemic preconditioning on activating HIF1a and VEGF expression.		
Li, China, 2023^22^	C57BL6 mice/ male/ 8-10wks/ 4 vs.4	70% WI (left+middle lobe)	None/gp78 gene OE	Liver/at the end of each treatment	RNA-seq/ Illumina	mRNA transcriptomics	gp78 OE I/R vs. WT I/R	Batched t-test, adjusted P-value, and fold change	1. Gp78-related DEGs were enriched in pathways linked to inflammation, protein ubiquitination, and lipid metabolism, highlighting their roles in liver IRI.	Tissue qRT-PCR, IHC, and WB	NA
		1. WI=1.5h, R=6h			Undefined	Proteomics			2. Proteomic revealed Gp78-related protein alterations were associated with ferroptosis and lipid metabolism. Gp78 OE upregulated lipogenesis genes like ACC1 and ACSL4, contributing to ferroptosis during liver IRI.		
					Undefined	Metabolomics			3. Metabolomic analysis revealed a significant increase in lipid metabolites and free fatty acids (FA) in Gp78 OE livers, with most lipids increased in Gp78 OE mice. Increased phosphatidylethanolamines (PEs) as polyunsaturated fatty acid (PUFA) chains were observed in Gp78 OE mice.		

Studies with rodent IRI models involving omics data were listed and ranked by publication date. ^a.^IPC represented I=10min and R=10min before ischemia, ^b.^IPO represented three circles of I=0.5min and R=0.5min after ischemia. Abbreviations: DE, differentially expressed; ER, endoplasmic reticulum; FDR, false discovery rate; I, ischemia; I/R, ischemia, and reperfusion; IHC, Immunohistochemistry; IPC, ischaemic preconditioning; IPO, Ischemic Postconditioning; IRI, ischemia and reperfusion injury; KEGG, Kyoto Encyclopedia of Genes and Genomes; KO, knockout; OE, overexpression; OI, Octyl itaconate; qRT-PCR, quantitative real-time PCR; R, reperfusion; TF, transcript factor; WB, western blot; WI, warm ischemia; WT, wild type.

**Table 2:** Main features of studies with omics application in rodent orthotopic liver transplant model

Author, country, publication year (ref.)	Species/gender/age or weight /number of cases vs. control	Animal model	Intervention/graft traits	Sampling/time	Assays/ platform	Omics	Comparison	Statistics	Major findings based on omics data	Validation	Database number
Wang, China, 2009^99^	Lewis rats/ male/12weeks, 250g/5 vs. 5	NA	None/none	Liver+serum/days 1-3-7 after LT	GC-MS (Agilent Technologies)	Metabolomics	Comparisons in post-transplant D1/D3/D7 and normal group	One-way ANOVA	1. Essential amino acids like L-threonine and phenylalanine decreased in serum but remained stable in the liver, likely due to surgical stress and liver metabolism.	NA	NA
									2. Post-transplant, metabolic states shifted left on day 1, approached normal on D3, and diverged by D7, linked to "seventh-day syndrome”.		
									3. Free fatty acids decreased in serum, while some liver metabolites remained stable, reflecting regulatory mechanisms.		
									4. Energy-related metabolites (e.g., fumarate, malate) present in the liver but not serum emphasize the importance of metabolomics in understanding post-transplant changes.		
Wu, China, 2009^98^	Lewis or BN rats/male/250g/4 vs. 4	Acute rejection	none/acute rejection	serum/1-3-7-10 days after LT	GC-MS (Agilent Technologies)	Metabolomics	Allogenic vs. Syngenic vs. normal control	one-way ANOVA	1. The PCA loading plot identified six key metabolites: glucose, hexadecanoic acid, L-threonine, proline, octadecanoic acid, and cholesterol.	NA	NA
		Lewis donors to BN recipients							2. In the allogeneic transplant group, decreasing glucose indicates impaired liver glucose regulation due to damage and immune rejection, serving as an early liver injury marker.		
									3. Increased fatty acids suggest fat mobilization triggered by metabolic disruption from immune rejection.		
									4. Elevated cholesterol links to liver damage and cholestasis.		
									5. Higher amino acids reflect protein breakdown and negative nitrogen balance from liver dysfunction, reducing rat survival rates.		
Qi, China, 2011^100^	Lewis rats/male/ 12weeks, 250g/5 vs. 5	Acute rejection	None/acute rejection	Plasma/day 30 after LT	GC-MS (Thermo Fisher)	Metabolomics	Allogenic vs. Syngenic vs. normal control	PCA	1. The syngeneic group was similar to the control group, whereas the allogeneic group was distinctly separated, highlighting significant metabolic differences between these transplantation models.	NA	NA
		Lewis donors to BN recipients							2. In the allogeneic group, cholesterol, urea, and L-aspartate levels increased, while levels of galactose, D-glucose, L-deoxyglucose, and gulose decreased.		
									3. In the allogeneic group, sugar reductions reflected impaired glucose regulation; amino acid elevations indicated stress and protein breakdown; increased urea and cholesterol suggest liver dysfunction and rejection.		
Yang, China, 2021^30^	SD rats/male/ NA/3 vs. 3	Steatosis	none/ steatosis	liver/24 hours after LT	BD Rhapsody system	Single-cell RNA sequence	HFD vs. control diet	Seurat package	1. Eleven cell types were identified, with significant differences in immune cell infiltration and proportions between normal donor livers and FLD.	Multiplexed IF	CRA004061
		HFD feeding 8 weeks							2. Myeloid cells in FLD exhibited a unique differentiation trajectory, ending with KCs and DCs, with distinct inflammatory and metabolic pathway activities.		
									3. A novel KC subtype (CSF3⁺ KC) was identified in FLD, characterized by high cytokine/chemokine expression and pro-inflammatory roles in fatty liver graft injury.		
									4.XCR1⁺ DCs, enriched in FLD, showed strong antigen-presenting ability and were linked to immune signaling pathways exacerbating liver injury.		
									5.Three T cell phenotypes were defined; in FDL, CCR7⁺ CD8⁺ T cells displayed pro-apoptotic and inflammatory traits, with notable CXCR4 and IL2RA changes.		
									6. Complex networks, particularly involving iKCs, XCR1⁺ DCs, and T cells, regulated immune responses and played key roles in FDL-associated liver injury.		
Liu, China, 2023^29^	Lewis rats/male/4 weeks/3 vs. 3	Steatosis	none/ steatosis	liver/before LT, 7 days, 4 months after LT	RNA-seq/Illumina Novaseq bp150	mRNA transcriptomics	MaS vs. non-MaS	limma package	1. DEGs associated with pre-LT-MaS were mainly enriched in PPAR signaling, mineral absorption, phagosome, and bile secretion.	qRT-PCR, WB *in vitro*	GSE193764
		MCD feeding 4 weeks			LC-MS/Thermo Fisher, UHPLC system	metabolomics	GF vs. non-GF	Batched t-test	2. DEGs related to post-LT-GF were enriched in DNA replication, cell cycle, and pyrimidine metabolism.		
									3. Post-LT-MaS were mainly enriched in pathways in glycine, serine, and threonine metabolism.		
									4. Differentially expressed metabolites associated with post-transplant GF were involved in aromatic amino acid metabolism.		
									5. E2F1-centered TRN played a crucial role in MaS-related GF.		
Yang, China, 2024^31^	C57BL/6 mice/NA/ 6 vs. 6	Steatosis	None/ steatosis	Liver/6 hours after LT	RNA-seq/Illumina Novaseq	mRNA transcriptomics	NC SHAM vs. HFD SHAM	Batched t-test	1. Proteomic analysis revealed differentially expressed proteins related to fatty acid metabolism and synthesis in the HFD LT group.	qRT-PCR, WB *in vitro*	NA
		HFD feeding 8 weeks			UHPLC coupled to an Orbitrap (Thermo).	metabolomics	NC LT vs. HFD LT		2. FABP4 was identified as a hypoxia-inducible protein in steatotic liver grafts to IRI.	IHC in tissue	
					UHPLC coupled to tims TOF Pro MS(Bruker Daltonics)	proteinomics	HFD SHAM vs. HFD LT		3. FABP4 inhibitors BMS-309403 can protect steatotic livers from IRI by reducing apoptosis and oxidative stress damage.		
							HFD LT vs. HFD BMS LT		4. The cAMP signaling pathway was enriched in steatotic grafts following FABP4 inhibitors utilization.		

Studies with rodent LT models involving omics data were listed and ranked by publication date. Abbreviations: ANOVA, analysis of variance; BN rat, Brown Norway rat; DC, dendritic cells; FLD, fatty liver donor; GC-MS, Gas chromatography/mass spectrometry; GF, graft failure; HFD, high-fat diet; IF, Immunofluorescence; IHC, Immunohistochemistry; KC, Kupffer cells; LT, liver transplantation; MaS, macrosteatosis; MCD diet, methionine and choline-deficient diet; NA, not available; NC, negative control; PCA, principal component analysis; qRT-PCR, quantitative real-time PCR; SD, standard diet; TOF, time-of-flight; UHPLC, Ultra-High Performance Liquid Chromatography; WB, western blot.

**Table 3:** Comparison of IRI and LT Models in Rodent Omics Studies

Feature	IRI Model	LT Model
Operation Complexity	Simple and controllable	Complicated and high expertise required
Damage Severity	Short-term, key damage in the LT process	Full process, including long-term complications
Recovery Time	Short	Long
Result Reliability	High, fewer confounding factors	Variable for immunological differences
Application	IRI mechanisms, short-term drug screening	Immune rejection, long-term outcomes, complex interactions

Abbreviations: IRI, ischemia-reperfusion injury; LT, liver transplantation.
